# A virtual-hybrid approach to launching a cardio-oncology clinic during a pandemic

**DOI:** 10.1186/s40959-020-00088-2

**Published:** 2021-01-13

**Authors:** Sherry-Ann Brown, Sahishnu Patel, David Rayan, Svetlana Zaharova, Mingqian Lin, Tarek Nafee, Bipin Sunkara, Ragasnehith Maddula, James MacLeod, Krishna Doshi, Joshua Meskin, David Marks, Jorge Saucedo

**Affiliations:** 1 Cardio-Oncology Program, Division of Cardiovascular Medicine, Medical College of Wisconsin, 8701 W Watertown Plank Road, Wauwatosa, WI 53226 USA; 2 Department of Medicine, Medical College of Wisconsin, Milwaukee, WI USA; 3 Medical College of Wisconsin, Milwaukee, WI USA; 4 Department of Medicine, Roger Williams Medical Center, Boston University School of Medicine, Providence, RI USA; 5 Division of Cardiovascular Medicine, Medical College of Wisconsin, Milwaukee, WI USA

**Keywords:** Cardio-oncology, Prevention, Telemedicine, Telehealth, Virtual care

## Abstract

**Background:**

As cardiovascular disease is a leading cause of death in cancer survivors, the new subspecialty of Cardio-Oncology has emerged to address prevention, monitoring, and management of cardiovascular toxicities to cancer therapies. During the coronavirus disease of 2019 (COVID-19) pandemic, we developed a Virtual-Hybrid Approach to build a de novo Cardio-Oncology Clinic.

**Methods:**

We conceptualized a Virtual-Hybrid Approach including three arms: information seeking in locations with existing Cardio-Oncology clinics, information gathering at the location for a new clinic, and information sharing to report clinic-building outcomes. A retrospective review of outcomes included collection and synthesis of data from our first 3 months (at pandemic peak) on types of appointments, cancers, drugs, and cardiotoxicities. Data were presented using descriptive statistics.

**Results:**

A de-novo Cardio-Oncology clinic was developed structured from the ground up to integrate virtual and in-person care in a hybrid and innovative model, using the three arms of the Virtual-Hybrid Approach. First, we garnered in-person and virtual preparation through hands-on experiences, training, and discussions in existing Cardio-Oncology Clinics and conferences. Next, we gleaned information through virtual inquiry and niche-building. With partners throughout the institution, a virtual referral process was established for outpatient referrals and inpatient e-consult referrals to actualize a hybrid care spectrum for our patients administered by a multidisciplinary hybrid care team of clinicians, ancillary support staff, and clinical pharmacists. Among the multi-subspecialty clinic sessions, approximately 50% were in Cardio-Oncology, 20% in Preventive Cardiology, and 30% in General Cardiology. In the hybrid model, the Heart & Vascular Center had started to re-open, allowing for 65% of our visits to be in person. In additional analyses, the most frequent cardiovascular diagnosis was cardiomyopathy (34%), the most common cancer drug leading to referral was trastuzumab (29%), and the most prevalent cancer type was breast cancer (42%).

**Conclusion:**

This Virtual-Hybrid Approach and retrospective review provides guidance and information regarding initiating a brand-new Cardio-Oncology Clinic during the pandemic for cancer patients/survivors. This report also furnishes virtual resources for patients, virtual tools for oncologists, cardiologists, and administrators tasked with starting new clinics during the pandemic, and innovative future directions for this digital pandemic to post-pandemic era.

## Introduction

Cardio-Oncology care has been adjusted in the COVID-19 pandemic with limited in-person clinic or hospital visits, increased use of teleconsultation, less frequent imaging, increased reliance on biomarkers, and considerations of differential diagnoses involving COVID-19 when evaluating cancer patients or survivors for possible cardiovascular toxicity [1]. Monitoring and management algorithms have been developed to help guide virtual care [2–4]. In the pandemic, we have changed the way in which we provide healthcare services at our clinics and institutions. This has challenged us to restructure current systems for the safety of our patients.

Various forms of innovation have come to bear in the pandemic, including telemedicine, digital health, artificial intelligence, social media, informatics, big data, and precision medicine [5, 6]. Telemedicine is the primary form of innovation that has been most developed in the pandemic [2, 5, 7]. Social media has been very helpful for dissemination of information, as well as education, and has been integral for creating online groups for support and determining the best ways for proceeding in the pandemic and advocating for our patients and colleagues in this period [5, 6]. In addition, the Doximity social media application has been valuable to practices across the nation, due to its telehealth platform (Doximity Video and Phone; https://www.doximity.com/dialer-video).

Despite the growing need, and allowances made during the pandemic, many centers do not have formal Cardio-Oncology clinics. Starting a new clinic can be challenging. The COVID-19 pandemic has made the process significantly more difficult, with the need to minimize exposure and maximize patient safety.

Currently, limited information is available on how to start a Cardio-Oncology Clinic during a pandemic, albeit given the high risk of morbidity or mortality in COVID-19-positive patients who also have cancer or CVD [8–13]. Several institutions have published on their experiences with starting in-person Cardio-Oncology clinics prior to the pandemic [8, 14–17]. One group has reported on their conversion from existing in-person Cardio-Oncology visits to telemedicine consultations, seeing 11 patients virtually within a few weeks [7]. Many have considered implications of the pandemic on the practice and study of cardio-oncology [2, 4, 5, 18–20], and two groups have suggested models for clinics converting from existing in-person care to televisits [4, 5]. Yet, no groups have directly addressed steps for de novo virtual-hybrid clinic formation within the limitations of the pandemic and without conversion of a pre-existing Cardio-Oncology clinic.

**Fig. 1:**
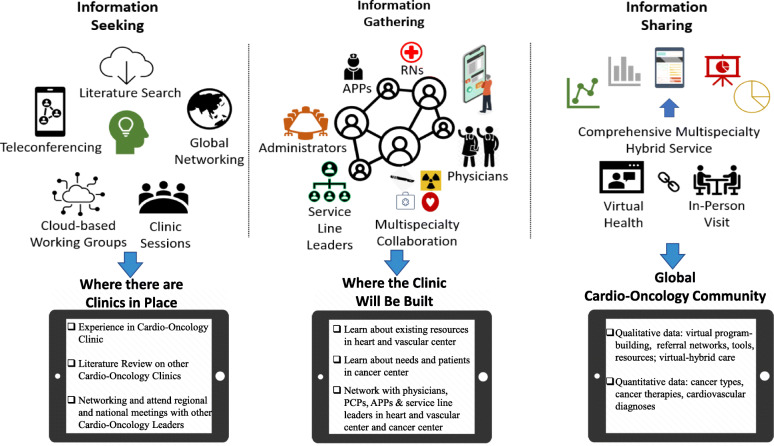
The Virtual-Hybrid Approach to Cardio-Oncology Clinic-building in the pandemic

Our report offers a template for other centers to develop their own new Cardio-Oncology clinics during the pandemic. We determined a Virtual-Hybrid Approach to clinic launch, with both virtual and in-person elements of three key arms: information seeking where there are existing Cardio-Oncology Clinics in place, information gathering where the clinic will be built, and information sharing to report on initial patient data demonstrating the success of the launch (Fig. 1). We then performed retrospective chart review to collect and synthesize data on the types of appointments (new versus established, virtual versus in-person), cancers (e.g., breast, prostate, leukemia, lung), cancer drugs, and cardiovascular toxicities (e.g., cardiomyopathy, hypertension) for patients seen virtually or in person in our new Cardio-Oncology clinic at Froedtert Hospital and Medical College of Wisconsin (F&MCW). Here, we will discuss our findings in the context of previous publications on launching Cardio-Oncology Clinics prior to the pandemic. Our results will present distributions of cancer drugs and types, and cardiovascular diagnoses, similar to previous publications on Cardio-Oncology clinic-building. However, we will differentiate and illuminate the techniques that leverage the virtual underpinnings of pandemic clinic-building. We submit that it is feasible to establish a new Cardio-Oncology Clinic for cancer patients or survivors with or at risk for cardiovascular toxicity from cancer therapy during a pandemic, providing optimal care for new patients in the midst of the need for safety and minimizing exposure. We also propose virtual resources for patients and clinicians and describe innovative future directions in the pandemic and post-pandemic period.

## Methods

### Virtual-hybrid approach

We pursued a Virtual-Hybrid Approach of information seeking, information gathering, and information sharing (Fig. 1). For information seeking, we focused on institutions that already had a Cardio-Oncology clinic in place. Prior to the COVID-19 pandemic, substantial experience was gained at an established in-person Cardio-Oncology clinic at a world-renowned leading hospital. Published manuscripts on pre-pandemic building and operations of Cardio-Oncology clinics at other institutions were reviewed. Extensive networking with leaders of Cardio-Oncology clinics around the United States and in the United Kingdom was accomplished at regional, national, and international meetings in person and on social media in a hybrid approach. These meetings were attended in person pre-COVID-19 and virtually during the pandemic. For information gathering, we addressed the location in which the new Cardio-Oncology clinic would be built. We learned about existing resources in the destination Heart & Vascular Center and existing needs and patients in the destination partner cancer center. We networked with physicians, advanced practice providers (APPs), nurses, service line leaders, and administrators in the Heart & Vascular Center and the cancer center, as well as in primary care and other supporting specialties. The subsequent Cardio-Oncology clinic sessions were intermingled with other subspecialty areas, to optimize availability for patient visits while filling clinic slots and tailoring spectrum of care to emerging patient needs. Cardio-Oncology patient visits were included in multi-subspecialty clinics.

### Retrospective study design

For quantitative data, we pursued a retrospective observational study to determine the distribution of outpatient visits in the first 3 months of our virtual-hybrid Cardio-Oncology Clinic. We reviewed data from charts of patients (all were 18 years of age or older) who received outpatient care from the new Director of Cardio-Oncology at F&MCW between April 15, 2020 and July 17, 2020 to determine which of these patients were considered to be in Cardio-Oncology, Preventive Cardiology, or General Cardiology. We collated all three to determine the percentage of patients seen in Cardio-Oncology, compared to the other two specialties. Preventive Cardiology was collated as a partner clinic to help build the Preventive Cardio-Oncology component of the Cardio-Oncology Clinic, to help apply established principles for prevention. From among the multi-subspecialty clinic sessions, we determined the proportion of patients who were specifically cancer patients or survivors with or at risk for cardiovascular toxicity from cancer therapy and thereby seen in the Cardio-Oncology Clinic. Next, we identified the distribution of cardiovascular toxicities in cancer patients or survivors seen in the Cardio-Oncology Clinic. We also evaluated the spectrum of cancer drugs received by cancer patients or survivors seen in the Cardio-Oncology Clinic. In addition, we summarized the types of cancers in patients seen in the Cardio-Oncology Clinic. Finally, we assessed the frequency of virtual visits during the course of the pandemic over our first 3 months for patients seen in the Cardio-Oncology Clinic. This retrospective review was approved by the F&MCW IRB; HIPAA informed consent was waived for this minimal risk study, which did not involve any form of intervention and was conducted in compliance with good clinical and research practice. The team designed and carried out the study with reliance on virtual communication tools.

### Data collection and analysis

Data gathering, management, and analysis were conducted at F&MCW. We collected patient-related, disease-related, treatment-related, and outcome-related data, particularly patient sex, type of appointment (new versus established, virtual versus in-person), type of cancer (e.g., breast, prostate, leukemia, lung), type of cancer drug, and type of cardiovascular toxicity (e.g., cardiomyopathy, hypertension). In order to minimize any risk of breaching patient confidentiality, all data collection occurred on institutional-based computing environments with de-identified data used for analyses. There were no alternative procedures for the subjects as this is a retrospective review of data that are not amenable to prospective collection and review. Descriptive graphs or tables of patient-, disease-, treatment-, and outcome-related variables distributions were prepared, with no comparisons made needing statistical tests.

## Results

### Virtual preparation

Preparation for starting the Cardio-Oncology clinic followed a Virtual-Hybrid Approach (Fig. 1, left). Five overarching factors employing virtual communication methods emerged to ensure the successful launching of the clinic. Team and individual experience and exposure to various areas of interest in Cardio-Oncology were achieved and assessed before and during the pandemic. Far-reaching connections to experts and potential collaborators in the field were developed and exercised. Close contact with the institution launching this clinic was important to determine the resources available; these resources dictated the strategy and potential outcomes of the clinic. Importantly, the expectations of others for the Cardio-Oncology clinic were determined and incorporated. Finally, recognition of the limitations that exist at the destination institution guided care and goal setting.

### Virtual inquiry

Before initiating the Cardio-Oncology clinic in the destination institution, existing structures, patient base, and needs in the Heart & Vascular Center as well as the Cancer Center were evaluated, adhering to pandemic protocols (Fig. 1, middle). Pre-existing building blocks for the planned Cardio-Oncology clinic were assessed, and the partner Preventive Cardiology clinic was investigated. We also evaluated characteristics of the cancer center patient population to best position the clinic for success.

### Virtual niche-building

Five main aspects of niche-building were pursued. Partnerships with Vascular and Cancer Center physicians, advanced practice providers, and service line leaders were developed to initiate and grow the clinic (Fig. 1, middle). The Cardio-Oncology team and clinic flexibility were demonstrated through openness to taking quicksteps. Presentations were made at Grand Rounds and rounds across the institution in Cardiology, Hematology/Oncology, Radiation Oncology, Surgical Oncology, Internal Medicine, and Family Medicine to promote the clinic capabilities. Collaborative solutions for problems facing fields complementing Cardio-Oncology developed trust and collaboration. Teamwork was developed by leveraging diversity of perspectives and virtual communication technologies, to establish effective patient care despite COVID-19 limitations.

### Hybrid care Spectrum

The hybrid F&MCW Cardio-Oncology Clinic was initiated and established in the outpatient setting, in close partnership with the Preventive Cardiology Clinic, Cancer Center, and inpatient Cardiology Consult and Hematology/Oncology teams (Fig. 1, right).

Initial and subsequent visits have been completed in person or by video, with phone visits also available for virtual return visits if patients without adept and available smartphone use have limited ability to appear in person. Virtual patient visits over our first 3 months occurred with the use of telemedicine platforms integrated with Epic (via MyChart for patients and Haiku/Canto for clinicians), or using the Doximity video call function. Patients with in-person appointments are screened appropriately on arrival for signs or symptoms of COVID-19 or exposure, following institutional protocols. Wearing masks is required of all patients, and each patient can be accompanied by a family member; some choose to also wear gloves or face shields. There is sufficient room for maintaining social distancing in the clinic waiting room and hallways.

### Innovation

**Fig. 2:**
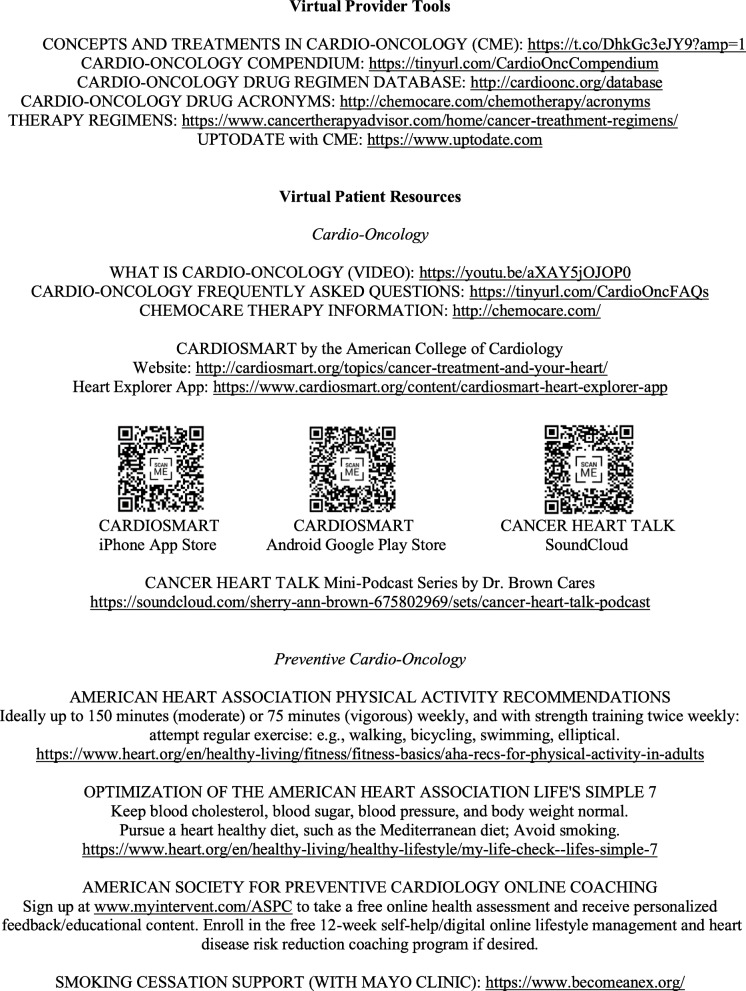
Virtual patient and clinician education and resources. Available online as PDF with hot links in the CardioOnc Compendium (https://tinyurl.com/CardioOncCompendium)

Current innovation in the clinic also includes Virtual Clinician Tools and Virtual Patient Resources (Fig. 2). For clinicians, the links for an AHA CME course on Novel Concepts, Current Debates and Treatment Considerations in Cardio-Oncology, an online Cardio-Oncology Compendium hosting risk assessment clinical decision aids, Cardio-Oncology Drug Regimen and Acronym Databases, and UPTODATE access for reviewing Cardio-Oncology drug information are supplied. For patients, the video from the International Cardio-Oncology Society explaining the Cardio-Oncology subspecialty, American College of Cardiology (ACC) mobile health (mHealth) CardioSmart education app and website, Cancer Heart Talk mini-podcast series accessed via SoundCloud app and website, Cardio-Oncology Frequently Asked Questions, and ChemoCare website are provided for patient-facing Cardio-Oncology and heart anatomy and physiology education, engagement, and awareness. Virtual Resources for Preventive Cardio-Oncology are also made available to our patients. These include the American Heart Association (AHA) Physical Activity Recommendations, AHA Life’s Simple 7 Webpages, American Society For Preventive Cardiology Online Coaching Webpages in partnership with Intervent, and the Become An Ex Smoking Cessation Support Webpages in partnership with Mayo Clinic. The resources are provided in the Epic patient portal MyChart, and more ways to make the resources accessible to a broad and diverse patient population are in development. Future innovation in the clinic will explore contemporary initiatives connecting patients and their safely guarded data with their permission with wearable devices, health information technology, informatics, artificial intelligence, personalized medicine, and additional mobile health (mHealth) applications.

### Virtual visit infrastructure and timeline

There was no pre-existing Cardio-Oncology program at the time of launching our de novo Virtual/Hybrid Cardio-Oncology Clinic. The newly recruited Director of Cardio-Oncology was tasked with the responsibility of launching the new clinic, with support from the Heart & Vascular Center clinic administrators, medical director, and Cardiology Division and Department of Medicine leadership. Prior to opening the Cardio-Oncology Clinic, the Heart & Vascular Center initiated virtual conversion then additionally collaborated with Inception Health (MCW’s innovation lab company) over the course of 4 weeks to iteratively develop the clinical informatics infrastructure for virtual visits. The video visits were designed to function using clinician’s personal smartphones, iPads, and tablets, with direct web browser video links from the electronic health record mobile application. Direct video calls through the Doximity mobile application were also approved. Existing Inception Health personnel re-allocated their time in order to adopt and maintain responsibility for the virtual component of all ambulatory clinics across the health system, in partnership with medical and administrative directors of each clinic area, such as the Heart & Vascular Center. No additional costs or hires were pursued to facilitate the development of the virtual visit infrastructure and timeline. Existing resources and personnel were re-allocated to virtual visit design to enable building the virtual clinics in the Heart & Vascular Center. To assist clinicians and billing compliance colleagues, note templates were created for video and phone visits to indicate patient informed consent for virtual visits due to the pandemic, as well as to capture limited appropriate physical examinations, in addition to the amount of time spent on records review and real-time medical counseling.

Initial consults were electronically triaged by either a cardiologist or a cardiology fellow supervised by a cardiologist. Each triage team determined which consults would be appropriate as virtual video visits, versus in-person to occur once the Heart and Vascular Center started re-opening routine physical visits, or whether patients needed to be evaluated urgently in person. During the first week of operation, the brand-new Cardio-Oncology Clinic started entirely virtually with only video and phone visits. As the Heart and Vascular Center re-opened for physical patient visits the following week, from week 2 through the remainder of the first 3 months the Cardio-Oncology Clinic had both virtual and in-person visits integrated throughout each clinic session weekly, based on whether patients were new and whether they had smart device or computer functionality available.

### Virtual referral network and process

Cardio-Oncology patient assessment begins within a referral network before the patient arrives at a Cardio-Oncology clinic. Consequently, forming a virtual referral network and enacting a user-friendly virtual referral process was a key component of building the Cardio-Oncology clinic during the pandemic. All referral patterns and networks for our de novo Cardio-Oncology Clinic were built from the ground up. Initial referrals were from within our health system; this quickly expanded to consults from outside of our health system encompassing the entire state. Patients were referred to our Clinic by clinicians or by self-referrals. Some of our local patients connected to us after being introduced to us by their clinicians in other states or through family members in other states who learned about us from their own clinicians or community-based Cardiology society outreach events.

Referrals across the institution and outside of our health system have come to us from the Divisions of Hematology and Oncology, Internal Medicine, Family Medicine, Surgical Oncology/Breast Clinic, and Survivorship Clinics. From the cancer center’s perspective, there may be many “triggers” that would warrant a Cardio-Oncology referral. For example, an abnormal ECG, an abnormal echocardiogram, cardiovascular symptoms, previous cardiovascular history (e.g., coronary artery disease, hypertension, cardiomyopathy) particularly in a patient who previously underwent treatment or is beginning new treatment with cardiotoxic neoplastic medications or radiation therapy and is at high risk of cardiovascular toxicity, or those in preparation for stem cell transplant, or oncologic surgery. Referral protocols were determined based on standard practice, discussions with colleagues in Medical and Radiation Oncology, Hematology, Bone Marrow Transplant, Surgical Oncology/Breast Clinic, Children’s Hospital, Radiation Oncology, Primary Care, and updated literature reviews.

Cancer survivors are at a higher risk than the general population for cardiovascular morbidity and mortality. If a cancer survivor needing to be evaluated is already under the care of a cardiologist, the referring provider can reach out to their cardiologist for guidance on the appropriate CV surveillance. If they do not already have a cardiologist, a Cardio-Oncology consult should be requested. The Cardio-Oncology consult can be placed using a direct Cardio-Oncology button within the universally available Cardiovascular Consult order panel. Referrers can also place a General Cardiology consult and mention the Cardio-Oncology physician by name as requested by the clinician or patient. An E-Consult functionality is also being implemented for those patients who need to be assessed sooner than the next available appointment, or for those patients who may not need a full Cardio-Oncology evaluation, or if referring providers are uncertain. The e-consult can also be placed as a second opinion requested by the inpatient Cardiology Consult team.

The inpatient Cardiology Consult service will continue to directly address inpatient consults from the inpatient hematology/oncology services. The inpatient Cardiology Consult service can collaborate with the Cardio-Oncology Clinic via formal Cardio-Oncology E-consults in the electronic health record Epic if a specific focused question arises regarding Cardio-Oncology relevant to the care of individual currently hospitalized patients that have already been formally evaluated by the inpatient Cardiology Consult service. After a patient has been formally evaluated by the inpatient Cardiology Consult service, if the patient is appropriate for outpatient follow up in the Cardiology clinic with Cardio-Oncology, this should be communicated to the primary Hematology/Oncology service. If appropriate at the time of consultation, the inpatient cardiology consult service can make the follow-up appointment. Oftentimes, this patient population remains in the inpatient setting for several weeks. If this is the case, the Cardiology clinic phone number and clinician information should be provided to the primary service to do so prior to the patient being discharged from the hospital.

### Virtual-hybrid multidisciplinary team

It is important to develop a multidisciplinary team and initially focus on allocation of pre-existing resources. Accordingly, some roles among our Cardio-Oncology clinic personnel are shared with other subspecialties. Our virtual-hybrid multidisciplinary Cardio-Oncology Clinic personnel include physicians, a nurse practitioner (NP), a nurse, a research support specialist, medical assistants, pharmacists, administrative assistants, and administrators. All personnel with pre-existing in-person roles and practices re-allocated a portion of their time to the development and practice of virtual visits.

Our clinic and partners consist of board-certified Cardiologists with special training in various cardiac subspecialties (e.g., cardio-oncology, preventive cardiology, heart failure and transplant, electrophysiology, interventional cardiology), who collaborate closely with our cancer experts. Our physicians together specialize in the prevention, diagnosis, and treatment of heart and vascular disorders resulting from side effects of cancer therapy. Our comprehensive team of advanced practice providers, nurses, and pharmacists work alongside our physicians to care for patients from the moment of cancer diagnosis through life’s survivorship journey. The NP typically sees established patients when needed to follow up on imaging, intervention, or diagnostic and management plans, and may also see select new patients. In complex cases, the NP discusses the care of established patients with both the cardio-oncologist and the referring clinician. The nurse assists with patient triage and communications (including addressing patient requests and queries), liaises closely with the nurse practitioner and pharmacists, and educates patients on Cardio-Oncology using virtual materials. Our clinical pharmacists function at the highest level of their advanced training, similar to all clinic personnel, and assist with medication education, review, titration, discussion, and prescription, particularly for heart failure, hypertension, hyperlipidemia, and smoking cessation, as well as commenting on potential drug interactions.

For Preventive Cardio-Oncology, we additionally partner with our dietitians and exercise physiologists to help advise our patients on nutrition and exercise plans, as well as our colleagues in cardiopulmonary stress testing where applicable. Further, in the pandemic, we provide patients with free online coaching options for lifestyle modification (Fig. 2). We also direct patients to AHA webpages with guidance on pursuing ideal cardiovascular health.

### Virtual-hybrid patient flow

**Fig. 3:**
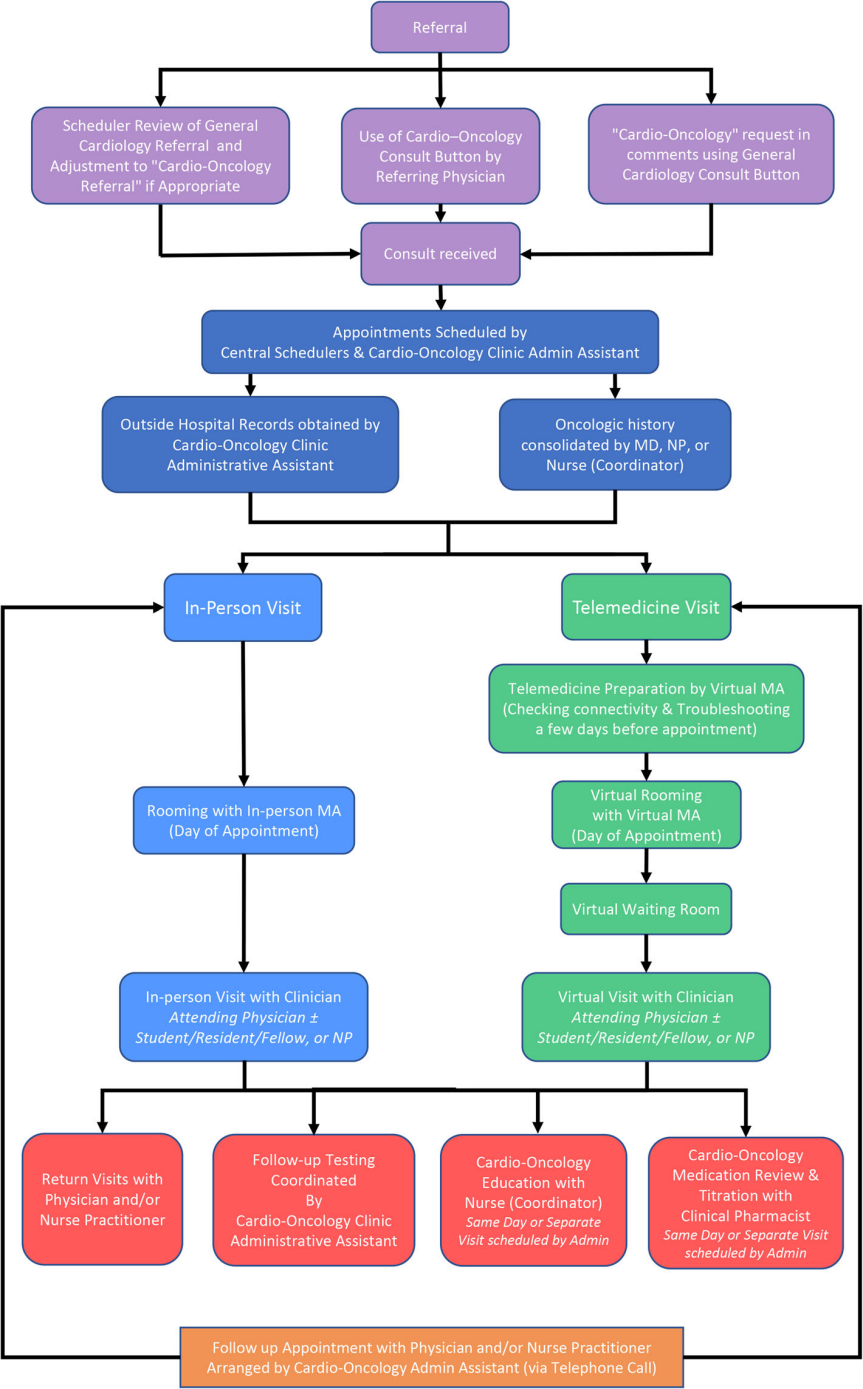
Virtual-Hybrid Patient Flow Chart. Admin = Administrative; MA = Medical Assistant; MD = Medical Doctor; NP = Nurse Practitioner

Once a referral is placed by the designated order buttons in the electronic health record, central schedulers or the Cardio-Oncology Clinic administrative assistant schedule the new patient for a video or in-person visit (Fig. 3). The clinic administrative assistant works closely with our health professionals in our interdisciplinary advanced subspecialty clinic to gather relevant clinical reports and history pertinent to patient appointments. Virtual medical assistants contact patients a few days before their appointments to confirm and troubleshoot virtual connectivity. On the appointment day, medical assistants then ‘room’ patients for virtual or in-person visits by preparing patients for their medical visits (including reviewing medications and in-person or at-home virtual vital signs), and also rechecking virtual connectivity for video visits. The clinician then completes the visit virtually or in-person and introduces the patient to the range of electronic resources available. Following the visit, the clinical administrative assistant arranges follow-up testing and appointments.

### Virtual risk assessment

**Fig. 4:**
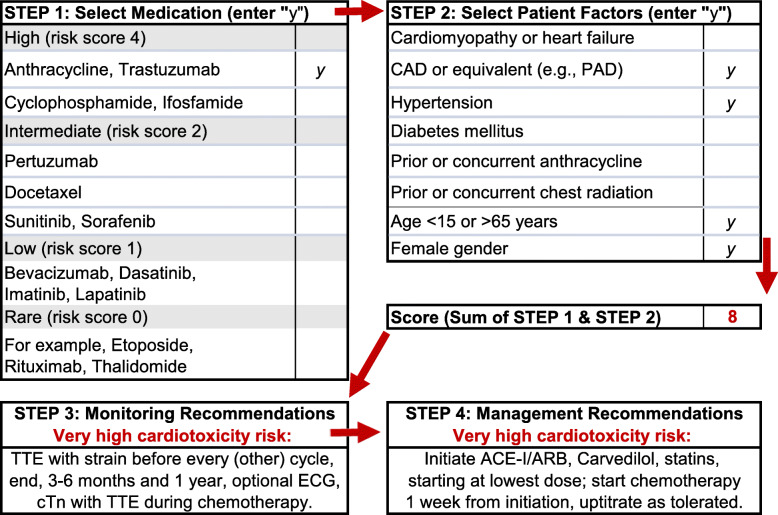
Virtual Cardio-Oncology clinical decision aid (CDA). Risk assessment (https://tinyurl.com/CardioOncCDA) to guide monitoring and management recommendations regarding development of cardiomyopathy for most Cardio-Oncology patients; a CDA specifically for women with early breast cancer is also available in the CardioOnc Compendium (https://tinyurl.com/CardioOncCompendium)

Baseline risk assessment and follow-up start with oncology and primary care [21]. Asymptomatic low risk patients with low-risk treatment plans can have continued assessment and follow-up by oncology and primary care in partnership. Patients who have symptoms, are at high risk based on their history, or are planned for high-risk treatment plans should be referred to Cardio-Oncology for prevention, monitoring, and management recommendations. Recommendations should adhere to society expert consensus, scientific statements, and guidelines for prevention, surveillance, and survivorship, and optimize CVD risk and medications [21]. A putative risk score based on medication-related and patient-related risk factors can be used to guide monitoring and management recommendations for most Cardio-Oncology patients [22], and can be used in a virtual clinical decision aid (https://tinyurl.com/CardioOncCDA) (Fig. 4). Specific risk scores are also available for adults treated with anthracyclines, trastuzumab, or other drugs, or for adult survivors of childhood cancers [9–13].

### Virtual management algorithms

Evidence-based management algorithms have been selected or developed as adjunctive resources for inpatient teams. They are available online in a virtual collection for use in the inpatient setting by the inpatient Cardiology Consult service or hematology/oncology teams to assist with diagnosis and treatment of cardiovascular toxicities from cancer therapies or cancer itself. The algorithms cover cardiomyopathy from anthracyclines or trastuzumab, planned chemotherapy with pre-existing cardiomyopathy, neurohormonal therapy or dexrazoxane for cardioprotection, myocarditis, persistent malignant pericardial effusion, hypertension, surveillance after radiation therapy or drugs that cause ischemia, malignant pericardial effusion, and other salient topics frequently encountered.

### Virtual community engagement

The local, regional, national, and international community was virtually engaged via social media posts on Twitter (using #MCWCardioOnc on @DrBrownCares or @PrevCardioOnc), podcasts hosted by the MCW CTSI (available on iTunes, Google, and Apple podcast platforms), Heart Success podcast series, and Cancer Heart Talk brief 15-min mini-podcast series (available on SoundCloud). Perspectives were also published for international community engagement in the Women Heart Alliance newsletter, as well as on the AHA Early Career Blog, ACC Women in Cardiology Blog, CardioOncTrain.Com Blog, and PrevCardioOnc.Com Blog. Virtual continuing medical education (CME) presentations were also given at the Wisconsin state ACC annual conference meeting, Midwest ACC annual conference meeting, Southeast ACC annual conference meeting, Brazilian Cardio-Oncology Symposium, and the Ohio State Cardio-Oncology CME conference, then subsequently at the AHA and ACC annual national scientific sessions.

### Distribution of patient data

**Fig. 5:**
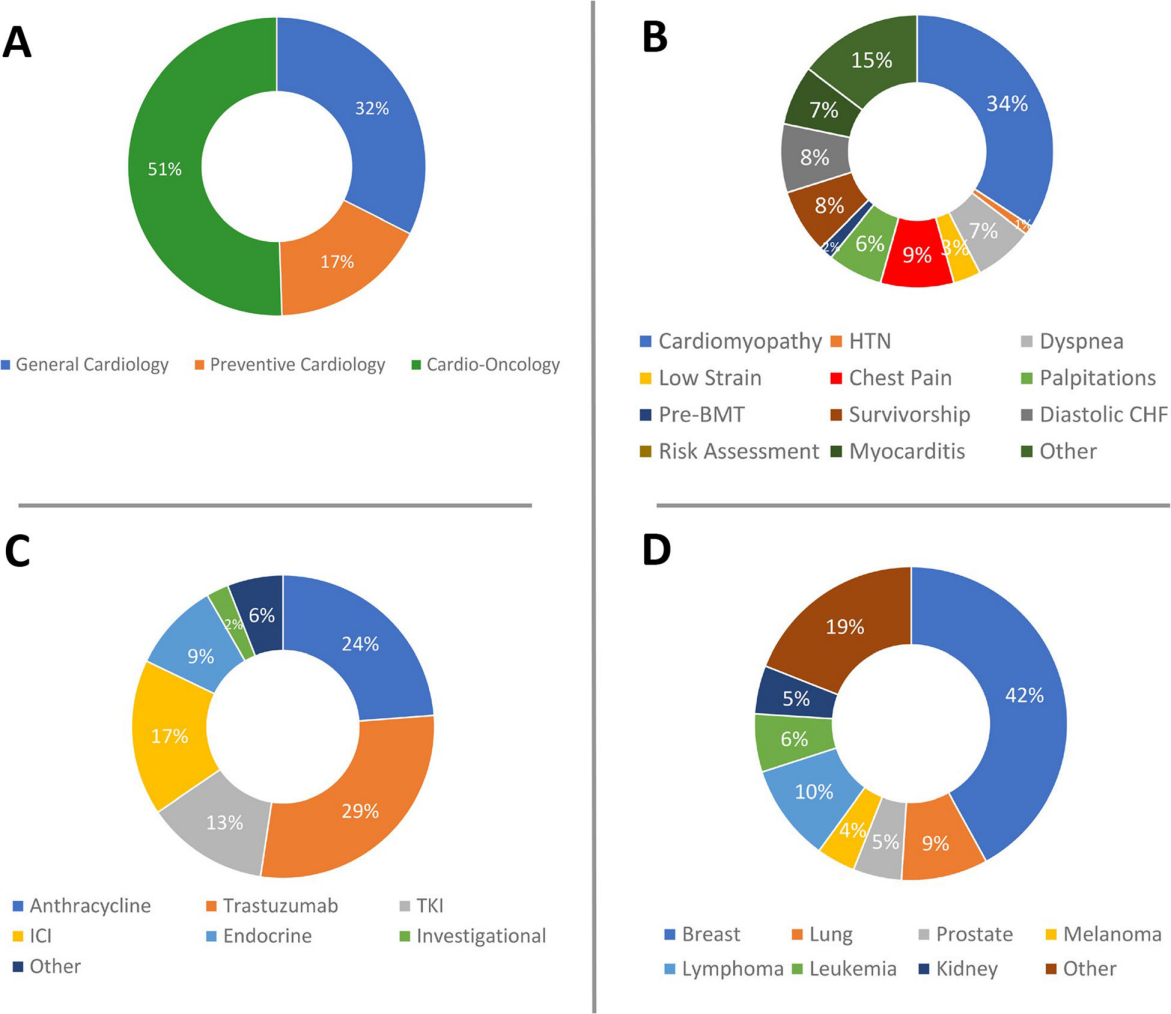
Initial Clinic-Building Outcomes Using the Virtual-Hybrid Approach. **a** Distribution of Cardio-Oncology, Preventive Cardio-Oncology, and General Cardiology patients seen in our multi-subspecialty clinic visits. **b** Distribution of cardiovascular diagnosis or indication for referral. **c** Distribution of cancer drugs. **d** Distribution of cancer types

In our multi-subspecialty clinic visits (virtual and in-person integrated and combined; *n* = 182; 136 new and 47 returns), approximately 50% of patient visits were in Cardio-Oncology, 20% were in Preventive Cardio-Oncology, and 30% were in General Cardiology (Fig. 5a). Overall among Cardio-Oncology visits, 65% were in person, consistent with early and safe clinic re-opening in a hybrid model, with 19% by video and 16% by phone, with the fraction by phone decreasing over time as patients and clinic personnel became more adept with troubleshooting video. Of new patients, 77% were in person, and the remainder by video. No Cardio-Oncology patients presenting in person developed any signs or symptoms concerning for COVID-19.

**Fig. 6:**
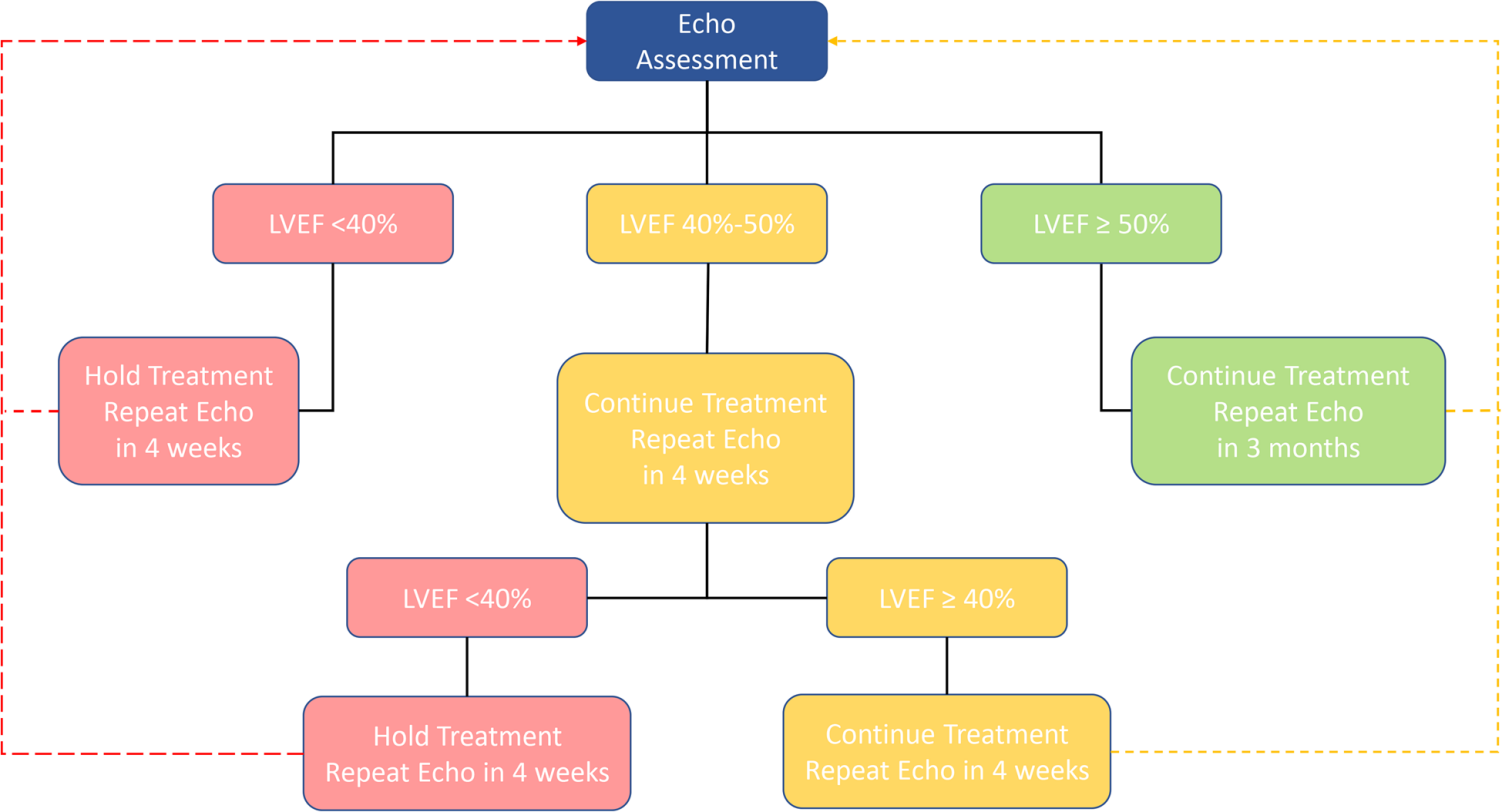
Algorithm for Continuation of Trastuzumab Therapy with Mild LV Dysfunction

The most frequent cardiovascular diagnosis or indication for referral was cardiomyopathy (34%) (Fig. 5b). Other diagnoses included decrease in global longitudinal strain, diastolic congestive heart failure, hypertension, myocarditis, dyspnea, chest pain, palpitations, survivorship, risk assessment, and pre-bone marrow transplant, among other cardiovascular diagnoses or visit indications. The most frequent cancer drug was trastuzumab (29%) (Fig. 5c), managed according to a novel algorithm developed in our de novo Virtual-Hybrid Cardio-Oncology Clinic based on the recent publication indicating the safety of continuation of trastuzumab for left ventricular ejection fraction of 40% or greater [23] (Fig. 6). The second most frequent cancer drug was anthracycline (24%). Other drugs included, tyrosine kinase inhibitors (TKIs), immune checkpoint inhibitors (ICIs), endocrine therapies, and investigational therapeutics, among others. The most frequent cancer type in our clinic was breast cancer (42%) (Fig. 5d). These trends in cardiovascular diagnosis or indication and cancer drugs or types were similar in assessments of virtual visits alone, with the most frequent being cardiomyopathy (43%), trastuzumab (41%), and breast cancer (44%), respectively. The findings of similar cardiovascular and cancer distributions in virtual versus in-person visits indicated an optimal qualitative return on resource and personnel investment.

### Imaging and medication titration

In our clinic, a distribution of cardiovascular diagnoses determines the imaging needed for each patient (Fig. 5b). Therefore, a number of imaging modalities are useful to our patients (e.g., echocardiography, computed tomography with or without angiography, magnetic resonance imaging, coronary angiography, myocardial perfusion imaging). Our most frequently used imaging modality is echocardiography. The frequency of obtaining echocardiograms has depended on each patient’s condition and cancer treatment. A substantial portion of patients coming to us on trastuzumab have needed an echocardiogram every 1–3 months, depending on the extent of adverse effects on left ventricular ejection fraction (LVEF) or strain In these patients, medication titration has occurred approximately every 2 weeks, and for very symptomatic patients with volume overload, they have often been seen weekly..

In our management algorithms, early referral prior to the onset of symptoms has been emphasized, especially in cancer patients or survivors with a history of cardiovascular disease, cardiotoxic neoplastic agents, or a high risk of cardiovascular toxicity. This has provided an opportunity for us to assess and discuss ways to optimize the benefit to risk ratio of continuing with the current cancer treatment plan, and more importantly how and when to put cardioprotective measures in place to facilitate safe cancer therapy. Such discussions have also resulted in closer monitoring. Some conditions have warranted proceeding to other modalities of non-invasive imaging, such as cardiac MRI if myocarditis is suspected. For cases in which coronary artery disease is suspected, our patients undergo functional assessment of their coronaries with a stress test or anatomical assessment with a coronary CT scan or invasive coronary angiography. In our practice, only exercise stress tests were halted due to the pandemic. Every other form of imaging including rest and stress echo, as well as MRI and nuclear medicine have remained readily available for those with cardiovascular toxicities or individuals considered to be at moderate or high risk. This allowed us to adhere to pre-pandemic imaging recommendations tailored during the pandemic to limit imaging if possible to those who are at higher risk for cardiovascular toxicities or who have already been diagnosed with these adverse effects [1–4] (e.g., Fig. 6).

## Discussion

The COVID-19 pandemic has inevitably compelled leaders of healthcare clinics to rethink and restructure approaches to deliver optimal care for patients. Our brand-new Cardio-Oncology clinic has been built to thrive in this new pandemic landscape by utilizing virtual technology as one of the key components of our clinic-building and care model since its inception. While existing clinics have reinvented their operations through the uptake of technology, our clinic has been able to capitalize on this resource to deliver virtual-hybrid care from the start. Virtual communication has proved useful to coordinate referral networks and care among providers within a multidisciplinary team across different clinics and departments. We see a variety of cancer patients, types, and drugs (Fig. 5), and our distribution results are generally congruent with reports from other leading cardio-oncology clinics [8, 14, 15, 17, 22].

Various methods have been developed for risk assessment to help guide providers and patients in determining the appropriate guidelines for care. We offer the use of virtual risk assessment tools such as the computed risk scores based on medication- and patient-related risk factors [22] (Fig. 4) (https://tinyurl.com/CardioOncCompendium), as well as recommendations for establishing cross-provider partnerships to continuously evaluate risk [21]. Other online databases containing useful information and guidelines are readily accessible and can help guide clinical practices. We encourage use of these virtual tools, which can further facilitate collaborative Cardio-Oncology care in the pandemic. Our conversations with international colleagues have suggested additional utility of these virtual tools beyond the pandemic. The online resources can be very helpful in settings where clinical practitioners work alone without support from nurses, pharmacists, nutrition specialists, or exercise physiologists.

Virtual-hybrid care has extended the care team’s capabilities for delivering and maintaining patient education and follow-up. The internet continues to be a robust resource, containing a wealth of health information that is easily accessible to the general population. Various mobile applications and electronic devices have also been developed in recent years to educate, track, and manage patients’ health and lifestyles. While these tools provide patients with greater accessibility and independence, they also create a valuable opportunity for healthcare providers to further engage patients. In a virtual-hybrid model, this becomes increasingly important, as patients may frequently transition between virtual and in-person visits. Forming care partnerships with patients through these virtual information and health-tracking resources becomes crucial in the continuity of care and proper health maintenance as we move through the pandemic.

Our most frequent cardiovascular diagnosis was cardiomyopathy (34%), which is reflective of the management need that first helped start the emerging field of Cardio-Oncology, and is similar to the most frequent cardiovascular diagnosis noted by clinicians from several other leading centers (20–35%) [24, 25]. However, Cardio-Oncology has grown remarkably over the last 10–20 years, with a wide spectrum of cardiovascular diagnoses and indications for referral (Fig. 5b) [8, 14]. Accordingly, at some other leading centers, the most frequent cardiovascular diagnosis or indication for referral has been reported as hypertension [14], arrythmia [17], or comprehensive risk assessment prior to beginning of therapy to optimize cardioprotection [8] in the practice of Preventive Cardio-Oncology [21]. This illustrates an opportunity for growth in our Clinic, to increase the fraction of high-risk patients who undergo comprehensive cardiovascular risk evaluation and management of risk factors prior to administration of cardiotoxic therapy.

Our clinic cares for patients with a range of cancer types (Fig. 5d). Individuals with breast, lung, and hematologic cancers represent a substantial proportion of our patient population, similar to other Cardio-Oncology clinics [8, 14, 22, 26]. The most frequent cancer diagnosis, breast cancer (43%), is consistent with reports from other leading cardio-oncology clinics such as the Mayo Clinic (39.2%) [15] and the Cleveland Clinic in Florida (44.3%) [14]. While hematologic malignancies such as leukemia and lymphoma represented 29% of our patients and was the second most prevalent cancer within our cohort, they comprised the most frequent forms of cancer at other cardio-oncology clinics such as at the Moffitt Cancer Center (31%) [8] and at UCLA (32.70%) [25]. However, the absolute difference was relatively insignificant. Overall, similar to these established cardio-oncology clinics, we receive patients from across a variety of cancers.

A wide breadth of cancer therapeutics is associated with cardiotoxicity [21]. Anthracyclines associate with cardiomyopathy, especially when used with trastuzumab. Targeted therapies (e.g., TKIs) can cause new or worsening of pre-existing hypertension. ICIs are associated with an increased incidence of myocarditis. Some cytotoxic chemotherapeutics, such as cisplatin, increase the risk of venous thromboembolism, and antimetabolites such as fluoropyrimidines have long been associated with a broad range of cardiotoxicities. Radiation therapy is associated with ischemic heart disease, valve dysfunction, conduction abnormalities, pericardial disease, and cardiomyopathy. Patients with cancer who have developed cardiovascular toxicity or who may be at high risk for cardiovascular toxicity should be referred to the Cardio-Oncology clinic for close follow-up.

The most frequently used cancer medication used among our patients was trastuzumab (29%), with the second most frequent being anthracyclines (24%). This was similar to other institutions, with anthracyclines and trastuzumab among the most common cancer drugs in their Cardio-Oncology clinics. Yet, anthracyclines were typically noted more commonly than trastuzumab. The Cleveland Clinic in Florida saw patients most commonly treated with radiation (40%), followed by anthracyclines (26.8%) [14]. The Moffitt Cancer Center most frequently had patients who were treated with anthracyclines (52%), with HER2 targeted therapies representing 27% of the cancer drugs [8]. This difference may reflect a high frequency of patients with HER+ breast cancer in our population (diagnosed by a ratio of HER2 to chromosome 17 signals on dual probe fluorescent in situ hybridization ≥2 or ≥ 6 HER2 signals/cell [27]), as well as the keen attention to a substantial fall in left ventricular ejection fraction or global longitudinal strain as a potential prognostic factor in our patients, per American Society of Echocardiography (ASE) guidelines [28].

All of these patient data distributions were obtained in the context of the Virtual-Hybrid patient flow in our de novo Cardio-Oncology Clinic (Fig. 3), that can be modeled by other future Virtual-Hybrid Cardio-Oncology clinics initiated during the pandemic. Table 1 compares the first few weeks of our de novo C-O clinic setup model with two published manuscripts describing conversion of pre-existing in-person C-O clinics to providing telehealth visits as an option for patients. The table shows similar numbers of patients seen in the initial periods of the clinics, although the numbers in our new clinic went from 0 to 10 in the first 3 weeks, compared to going from up to 40 patients weekly to 11 patients in 2.5 weeks for a group that converted their in-person clinic to a virtual option. Overall, CV diagnoses and cancer types were comparable; distributions of cancer drugs were not reported by the other group. Important differences were noted. Most of our patients in the pandemic were new (90%), given the de novo status of the Cardio-Oncology Clinic, while the converted virtual clinic of another group initially focused on established patients for > 50% of their patient visits. While key personnel were also the same (e.g., physician, advanced practice provider, nurse or nurse coordinator), we also report virtual versions of supportive staffing patterns, including the virtual scheduling and rooming process and pharmacy and lifestyle modification visits. Additionally, trainees have been integrally involved in the establishment of our Cardio-Oncology Clinic, with residents training in program-building, and medical students and fellows training in ambulatory cardio-oncology clinical practice and cardio-oncology critical thinking, respectively. Finally, besides the patient flow (Fig. 3) and de novo nature of our Virtual-Hybrid clinic initiated in the pandemic, our unique contribution may be the virtual resources, compared to the essential “webside manner” [7] or an alternative algorithm [4] for triaging virtual or in-person visits to the physician or advanced practice provider (Table 1).

**Table 1:** Comparisons Among Clinic Models Described In The Pandemic

	Virtual-Hybrid Clinic	Telehealth Clinic (14)	Triage Clinic (4)
Model Type	De Novo	Conversion	Conversion
Time Frame Compared	3 weeks	2.5 weeks	Not reported
Number of Patients	10	11	Not reported
New Visits (%)	90	45	Not reported
Variety of CV Diagnoses	Yes	Yes	Not reported
Variety of CA Types	Yes	Yes	Not reported
Variety of CA Drugs	Yes	Unknown	Not reported
Referrals	Yes	Unknown	Yes
Scheduling	Yes	Unknown	Not reported
Rooming Process	Yes	Unknown	Not Reported
Virtual AA	Yes	Unknown	Not Reported
Virtual MA	Yes	Unknown	Not Reported
Virtual Physician	Yes	Yes	Yes
Virtual APP	Yes	Unknown	Yes
Virtual Pharmacist	Yes	Unknown	Not Reported
Virtual Nurse (Coordinator)	Yes	Unknown	Yes
Unique Contribution	Virtual Resources	Webside Manner	Triage Algorithm

Similar to the formation or conversion of Cardio-Oncology clinics, many protocols for treatment regimens and cancer patients are yet to be standardized. While no standard protocols have been widely adopted at Cardio-Oncology practices, various institutions and writing groups have proposed some approaches (e.g., ASE or ASCO guidelines). We have collaboratively developed institutional algorithms for various cardiovascular toxicities and medications based on existing scientific statements, society guidelines, expert consensus statements, and manuscripts from leading cardio-oncology research institutions. The goal is to adopt, adapt, develop, and continuously update these algorithms, as new literature arises in order to establish best practices and an institutional standard of care.

## Conclusion

Starting a new Cardio-Oncology Clinic in the pandemic has its challenges, and yet for our patients can be invaluable. Appropriately competing priorities in the pandemic can limit the scheduling of meetings and gathering of people together in one virtual room to discuss a mutual vision. Gathering resources for patient and clinician education can also be formidable, as can social distancing and obtaining important imaging. However, multiple virtual one-on-one or small group meetings can be beneficial for building institutional relationships. Similarly, virtual visits have risen to the challenge to ensure maintenance of patient care throughout the pandemic. Modifications have also been made to enable safety and distancing during imaging. With the benefit of these adjustments to address the challenge, this report provides a foundation for initiating a cardio-oncology clinic in the pandemic, with virtual resources and tools to equip patients and clinicians.

In the future, we will also lay out a roadmap for initiation of comprehensive cardio-oncology programs with the five pillars of patient care, education, research, community engagement, and innovation in the era of digital transformation accelerated by the pandemic. Novel risk modifiers and risk attenuation methods, such as breast arterial calcification, clonal hematopoiesis of indeterminate potential, and Cardio-Oncology prehabilitation, habilitation, and rehabilitation will also be addressed. Future innovation to implement recommendations from clinical trials across the nation currently underway that utilize mobile health or web-based diet and physical activity interventions and/or seek to determine the impact of cardioprotective pharmacotherapy in Preventive Cardio-Oncology will also be assessed (ClinicalTrials.Gov: NCT01988571, NCT02943590, NCT02562716, NCT01968200, NCT03265574, NCT03760588, NCT03386383, NCT02244411, NCT03223753). Many of these studies incorporate virtual technologies that will be very helpful during and after the pandemic as we continue pursuit of digital transformation.

## Data Availability

The datasets during and/or analyzed during the current study available from the corresponding author on reasonable request.
